# 3D-Printed Patient-Specific Casts for the Distal Radius in Children: Outcome and Pre-Market Survey

**DOI:** 10.3390/ma15082863

**Published:** 2022-04-13

**Authors:** Simone Lazzeri, Emiliano Talanti, Simone Basciano, Raffaele Barbato, Federico Fontanelli, Francesca Uccheddu, Michaela Servi, Yary Volpe, Laura Vagnoli, Elena Amore, Antonio Marzola, Kathleen S. McGreevy, Monica Carfagni

**Affiliations:** 1 Meyer Children’s Hospital, Viale Gaetano Pieraccini, 24, 50139 Florence, Italy; emiliano.talanti@meyer.it (E.T.); simone.basciano@alice.it (S.B.); raffaele.barbato@meyer.it (R.B.); federico.fontanelli@meyer.it (F.F.); laura.vagnoli@meyer.it (L.V.); elena.amore@meyer.it (E.A.); kathleen.mcgreevy@meyer.it (K.S.M.); 2 Department of Industrial Engineering, University of Florence, Via Santa Marta 3, 50139 Florence, Italy; francesca.uccheddu@unifi.it (F.U.); michaela.servi@unifi.it (M.S.); yary.volpe@unifi.it (Y.V.); antonio.marzola@unifi.it (A.M.); monica.carfagni@unifi.it (M.C.)

**Keywords:** 3D printing, pediatrics, customized implants, orthopedic device, orthosis modeling, reverse engineering, personalized medicine

## Abstract

Background: Orthopaedic and Trauma surgery is expected to undergo profound transformation as a result of the adoption of 3D technology. Among the various applications, patient specific manufacturing of splints and casts would appear to be, particularly in children, an interesting implementation. This study aims to assess the safety of patient specific 3D casts obtained with a newly developed 3D-scanning devise in a small case series. We therefore conducted a clinical outcome and pre-marketing study in 10 consecutive patients with distal radius fractures treated at an Academic Level I Pediatric Trauma Center. After the application of the 3D cast, patients underwent three consecutive evaluations in the following 21 days. The main outcome measurements were: pain, skin lesions and general comfort, and acceptance of the cast. The three domains were measured with the Visual Analogue Scale (VAS), the NPUAP/EPUAP classification and the Positive affect-Negative affect Scale for Children (PANAS-C), the Self-Assessment Manikin (SAM) clinical psychology tests and a Likert-type five item questionnaire, respectively. A final mechanical analysis of the cast was carried out to confirm product integrity. Results: The results obtained were consistently positive in the investigated domains of general comfort, efficacy of contention and mechanical integrity of the 3D-printed cast as well as in the practicability of the supply chain. Conclusions: This study provides Level IV evidence that patient specific 3D printed casts obtained with a specifically designed software were safe in the management of “buckle” fractures of the distal radius in children. These results encourage to extend the technology to the treatment of more demanding fractures.

## 1. Introduction

In recent years, the rapid emergence of 3D printing technology supported by reverse engineering (RE) techniques has enabled a wide variety of biomedical applications [1,2]. Orthopaedic and Trauma surgery is one of the medical disciplines expected to undergo a profound transformation as a result of the adoption of this technology [3,4]. For example, 3D printing can be used with magnetic resonance imaging (MRI) and computed tomography (CT) to reproduce patient-specific anatomic models and help in preoperative planning. Three-dimensional printed models of fracture configuration or pathology can allow surgeons to visualise relevant anatomy and help in executing complex operations, such as pelvic trauma surgery [5]. Furthermore, tangible 1:1 printed 3D models are helpful in measuring screw lengths, choosing fixation devices and pre-contouring (pre-shaping) fixation plates, especially when facing a complex fracture pattern [6]. Customised implants that match the patient’s anatomy can be manufactured and can be used for direct replacement of a large defect, as may be the case after tumor resection when a reconstruction in limb-salvage surgery is planned [7,8]. Finally, patient-specific instrumentation can be printed in 3D and may help with precise implant placement.

Among the many applications of 3D printing, patient-specific manufacturing of splints and casts appears to be, particularly in children, an interesting implementation. There are, however, some technical problems that need to be addressed to achieve this result. The manufacturing of a 3D printable cast follows the three typical steps of the RE framework: (i) acquisition of the specific anatomy in the form of 3D data; (ii) modelling of the medical device within a CAD environment; (iii) manufacturing the device with addictive techniques. The acquisition process involves the conversion of digital imaging and communications in medicine (DICOM) format data derived from MRI or CT or the collection of the desired segment anatomy with optical scanners. Obtaining data from MRI and CT scans to produce patient-specific splints is unpractical and has ethical and medico-legal implications, and is highly arguable, especially in paediatrics. On the other hand, optical scanning technologies need to be both inexpensive and fast. Indeed, a quick acquisition process avoids a prolonged immobilisation for the patient with a fractured limb, thereby minimising movement artefacts and improving the patient’s compliance. The modelling of the device through CAD software is another critical point. To make the technology attractive in a clinical setting, a CAD modelling process designed to be handled by non-experts seems a desirable feature. Both topics were the subject of previous studies, specifically, from the initial definition of a reliable and repeatable strategy to designing the CAD model of the patient-specific orthosis that could be used without alteration for each new case [9], an acquisition infrastructure was realised along with a semi-automatic modelling procedure of the customised device [10]. In addition, to verify the user-friendliness of the system, thus the possibility of being used by non-experts in the field of 3D acquisition and modelling, usability tests were carried out [11].

The recent literature offers some experiences on 3D-printed, patient-specific cast manufacturing [12,13,14], but few are clinical trials, none of which focus on the paediatric population [15,16]. This study, carried out through multidisciplinary collaboration between the Meyer’s Children Hospital of Florence and the Department of Industrial Engineering of the University of Florence, aims to assess the applicability of 3D patient-specific casts obtained with a specifically designed integrated system [11] and to test its clinical safety in a small series of children with distal radius fracture.

## 2. Methods

The study was conducted at an Academic Level I Paediatric Trauma Center. Formal approval from the local Ethical Committee and from the Minister of Health was obtained (Comitato Etico Regionale per la Sperimentazione Clinica della Regione Toscana, Sezione: CEP, reference number 117/17).

From 1 October to 18 December 2018, ten consecutive children affected by a “buckle” fracture of the distal radius were enrolled. Inclusion criteria were: (1) age from 7 to 13 years; (2) isolated “buckle” fractures. Exclusion criteria were: (1) other fracture patterns; (2) presence of other fractures or trauma; (3) history of acute or chronic illness; (4) presence of any skin affection equal or exceeding grade 1 according to the NPUAP/EPUAP classification; (5) history of any reaction or hypersensitivity that could be relatable to the material employed to produce the splint. Possible reasons for withdrawal from the study were: Visual Analog Scale (VAS) > 7 at any time; skin lesion > 1 according to NPUAPA/EPUAP at any time; loss of immobilisation due to any observable evidence of device failure.

Children were enrolled after that parents were informed of the purpose and the objectives of the study and agreed to a specific written consent.

Scanning of the forearm was performed in a space next to the Emergency Department. After scanning, the forearm was provisionally immobilised in a plaster slab. An appointment was arranged within 24–48 h after enrolment for each patient in the outpatient clinic to have their plaster slab replaced with the 3D printed cast.

**Figure 1:**
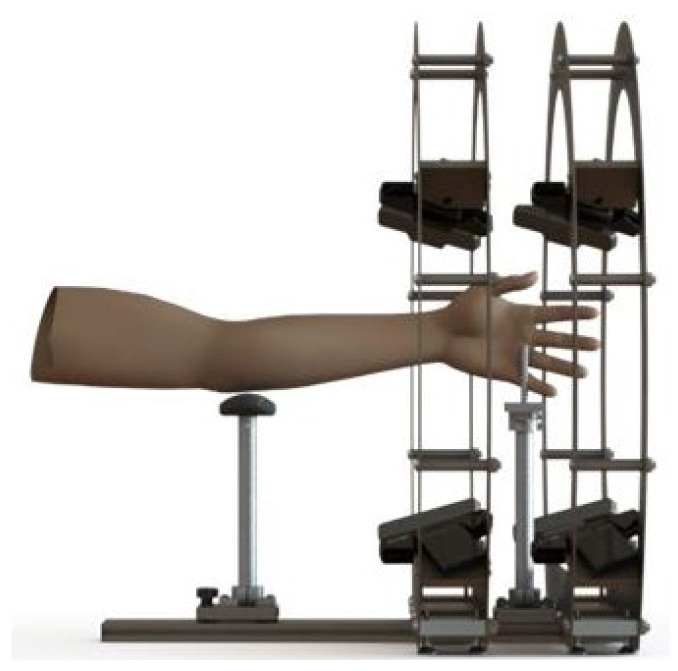
Layout of the instant hand wrist arm 3D scanner.

The 3D anatomy acquisition phase was performed with a specifically designed optical scanner composed of eight Intel RealSense SR300 digital cameras assembled on a circular frame (Figure 1). The digital cameras are CE-certified for the acquisition of 3D images and approved by the FDA as *Laser I products* in conformance with EN/IEC 60825-1 2007 Safety of Laser products.

**Figure 2:**
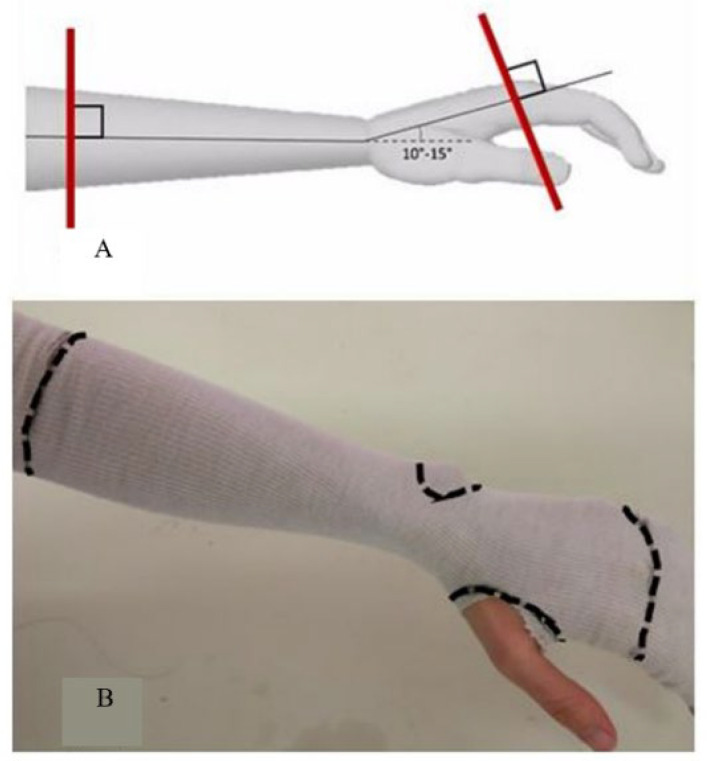
Neutral “thumb-up” position with the wrist in slight extension (10–15°) (**A**). The base of the thumb, the head of the metacarpals and the ulnar styloid positions are then marked by the operator (**B**).

The 3D acquisition was obtained with the forearm positioned on a frame specially designed to hold the wrist in the neutral “thumb-up” position with the wrist in slight extension (10–15°). Sliding elbow support is capable of accommodating significantly different anatomies, as is likely to occur in a paediatric setting. Prior to scanning, a Lohmann & Rauscher Tg^®^ Soft stockinette was applied on the forearm and points 3 to 5 cm distal to the elbow flexion crease, the base of the thumb, the head of the metacarpals and the ulnar styloid positions manually marked (Figure 2).

On the acquired file, the previously marked positions were identified, and portions of the 3D model were eliminated. Three-dimensional data manipulation was achieved with a specifically designed software (T3DDY-Oplà) [11] which allows preparing a file that permits the design of a patient-specific cast according to the defined medical constraints.

**Figure 3:**
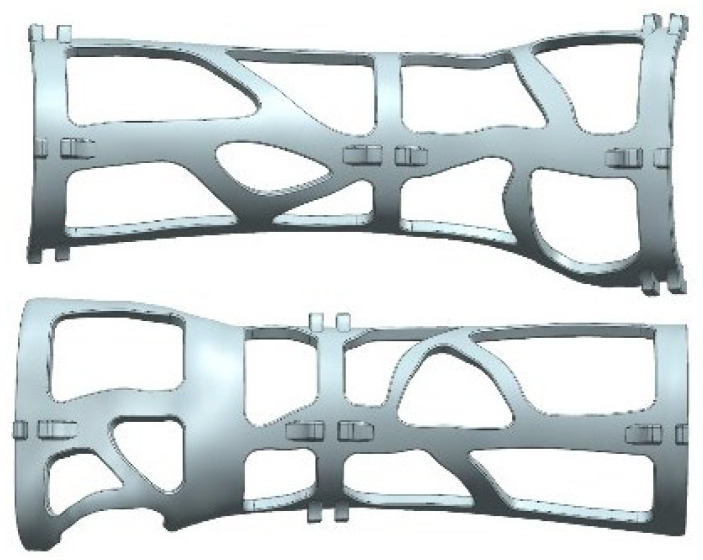
Example of a final model composed of two shells with ventilation holes and bend-ties housings.

The orthosis CAD modelling procedure was carried out within Siemens NX modelling environment [11] in which, as the last step, holes for ventilation and weight reduction were designed. The final model was composed of two shells, each one equipped with bend-ties housings for easy application and fixing. At the end of the modelling procedure, the digital model was exported into two distinct STL files (one for each part of the device), ready to undergo construction in an additive manufacturing process (Figure 3).

The cast was printed using a professional FDM (i.e., Fused Deposition Modeling) 3D printer, the 3D Stratasys F370 printer, in a laboratory located outside the hospital. The material chosen to construct the cast was acrylonitrile butadiene styrene (ABS), already approved by the FDA for a similar casting device known as ActivArmor^®^ [17]. The final cast consisted of two shells connected with three plastic ties positioned at the extremities of the cast and at the wrist.

**Figure 4:**
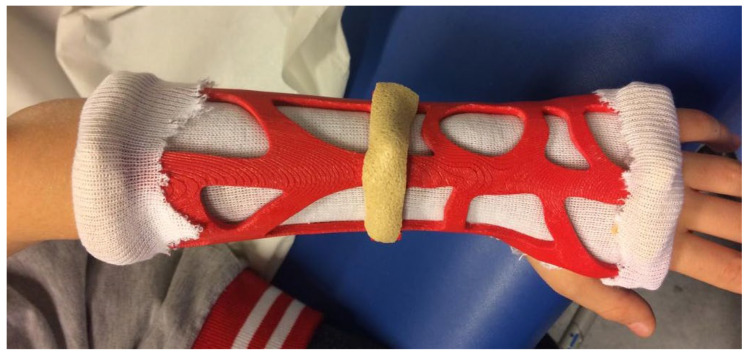
The 3D-printed orthosis positioned.

The 3D printed cast was fitted to the patient at a prearranged outpatient clinic appointment (Figure 4).

After applying the cast, patients were re-evaluated after 3, 7 and 21 days in the outpatient clinic. At each follow-up visit, general comfort was evaluated, a visual inspection of the cast was undertaken, and any possible structural anomaly was described.

The cast was always removed to enable visual inspection of the skin and then reapplied. At the latest evaluation at 21 days, a final orthopaedic evaluation was performed. Primary outcomes were: general comfort, the efficacy of contention and mechanical integrity. A secondary outcome was the practicability of the supply chain, which is the availability of the 3D printed cast within the planned timeframe of 24–48 h from data acquisition.

**Figure 5:**
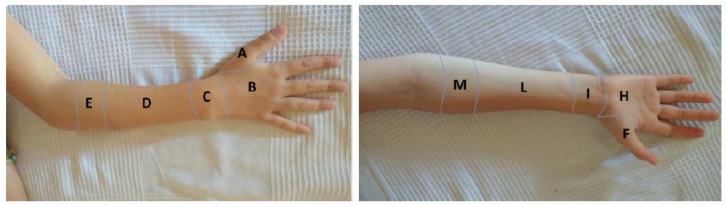
Definition of location/anatomic landmark to identify possible pain sites.

General comfort was assessed as follows: pain during treatment assessed by location (Figure 5) and intensity as measured with the Visual Analogue Scale (VAS) [18]; the presence of skin lesions according to NPUAP/EPUAP classification [19]; overall wellbeing according to the Italian adaptation [20] of Positive affect (PA)-Negative affect (NA) Scale for Children (PANAS-C) [21] and the Self-Assessment Manikin (SAM) [20] clinical psychology tests. Representative mean scores were extracted from available normative values [20].

The SAM is a nonverbal pictorial assessment technique that measures pleasure, arousal and dominance associated with a person’s affective reaction to a wide variety of stimuli for children aged 3 to 16 years [22,23,24].

Other features related to general comfort were explored by submitting to children and parents a five items questionnaire constructed according to a Likert-type scale. The first three questions were related to the activity level, and the possible answers were rated on a scale 1 to 5, with “1” being the worst answer (never) and “5” the best one (as usual); one question was related to the presence of itch, and the possible answers were rated on a scale from 1 to 5 being “1” no itch and “5” very itchy; the last question was related to the overall opinion on the cast, with possible answers ranging from “1” very poor, to “5” very good. This short questionnaire was administered to children and parents at each follow-up appointment.

Differences in general comfort were assessed using medians with the Mann–Whitney U test for independent variables.

Clinical efficacy of contention was assessed at the time the 3D-printed cast was applied. Inspection of the forearm-cast construct was checked at anatomic landmarks. Tolerance between the cast and the stockinette could not exceed 3 mm. Moreover, as mentioned above, a standard clinical evaluation was performed by an experienced orthopaedic surgeon (SL) at the end of treatment. This evaluation consisted of a general inspection of the limb, range of motion measurement after cast removal, tenderness evaluation over the fracture site and evaluation of the need for further treatment.

The mechanical integrity of the cast was assessed at the end of the treatment by performing a 3D scan on every single cast in order to compare the newly generated file with the original orthosis file. Since the structural strength of the device was assured in a previous study [25], in this work, it was decided to only test if the device had undergone major deformations after being used for treatment. For this reason, only a deviation test between the initial device and the device scan at the end of the treatment was performed.

As reported above, an analysis of the supply chain was conducted with special consideration for the time interval between the transmission of the cast file and its availability for application. Possible flaws for the delay in supply were noted.

## 3. Results

Ten consecutive children with “buckle” fractures of the distal wrist were enrolled from 10 October to 17 December 2018. All cases were admitted and screened for inclusion criteria at the Accident and Emergency Department of the Meyer Children’s Hospital in Florence. The mean age was 8.9 years (7–10 years), and there was an equal distribution between males and females (5:5). The right side was affected in six cases.

At first evaluation after three days, pain was reported in three patients: two complained of pain at the fracture site (VAS 2) and one at the ulnar styloid (VAS 3). Pain was never reported at seven days. On the final evaluation at 21 days, one patient complained of pain at the proximal end of the cast (VAS 1), one at the base of the thumb (VAS 2), one at the hypothenar eminence (VAS 2) and one at mid-forearm (VAS 3) (Table 1).

No skin lesion was detected at any time according to the NPUAP/EPUAP classification. Two cases of mild irritation at the base of the thumb were noted. The irritations disappeared after slight manual smoothening and padding of the cast.

The PANAS-C scores for PA and NA were, respectively, 55.47 ± 9.6 and 28 ± 2.4 for girls and 45.64 ± 10.5 and 23.19 ± 5.2 for boys. The normative scores for PA and NA are, respectively, 42.99 ± 8.1 and 27.38 ± 8.2 for girls and 42.03 ± 8.3 and 25.84 ± 7.6 for boys.

The mean SAM scores were 3.9 ± 0.1 for pleasure, 1.6 ± 0.2 for arousal and 3.6 ± 0.2 for dominance (Table 2). 

**Figure 6:**
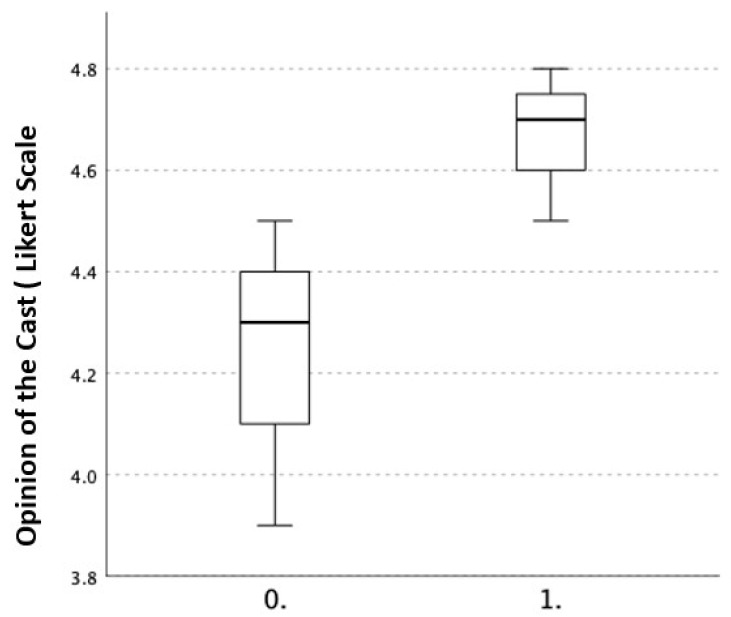
General acceptance of the cast: 0 = children; 1 = parents. Difference not statistically significant.

All answers to the evaluation questionnaire ranged in the positive scores, both in children as well as in parents, and the median scores were generally overlapping. No statistically significant differences were observed. Mean scores for each item at each evaluation are reported in Table 3. Interestingly, parents reported a higher overall opinion of the cast, although not statistically significant (Figure 6).

Results on the efficacy of contention showed fitting within 3 mm tolerance in seven cases, while in three cases, the tolerance measured between 3 and 5 mm. The clinical evaluation at the end of treatment never revealed skin lesions, tenderness at the fracture site or elsewhere, or limitation of the range of motion as evaluated several minutes after cast removal. Further treatment was never indicated.

### Mechanical Integrity

**Figure 7:**
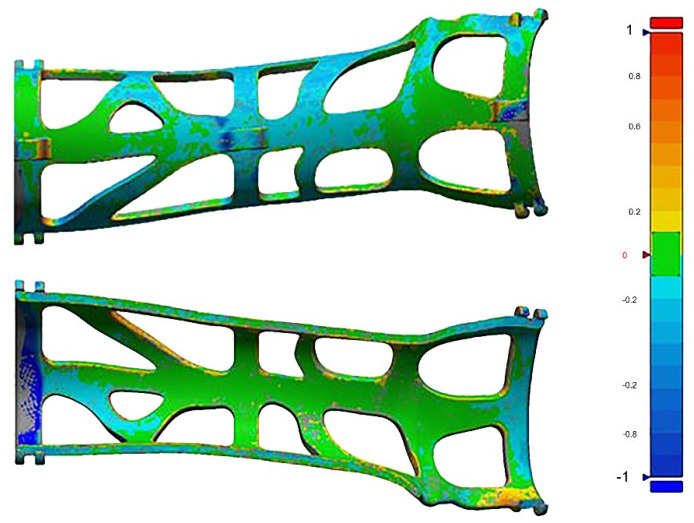
Mechanical integrity: comparison between the 3D scanning of an orthosis at the end of treatment with the related original 3D file. Bar on the right shows deformation in millimeters from original file; green is no deformation, red +1 mm and blue −1 mm.

No permanent deformations were measured after 3D scanning of the used orthosis. The averaged measured error between the original cast 3D file and the acquisition of the used 3D printed cast, over the 10 examined cases, was 1 mm with a maximum error of 2 mm, considering the absolute minimum and maximum values (deviation values are shown in Table 4). This confirmed that all casts remained intact. An example of the comparison between the used and original orthosis 3D file is reported in Figure 7.

The supply chain allowed the final cast to be available for application within 24 h from data acquisition to cast availability. The final price of a single 3D splint was EUR 30, which is undoubtedly higher than the traditional cast or splint, but about the same price as a common industrial removable brace in our region. The final price does not take into account a possible amortisation of the scanner (estimated price EUR 3500), the 3D printer and the price of the essential software.

## 4. Discussion

This study provides Class IV evidence that a 3D printed orthosis can be effective, safe and well-tolerated in treating “buckle” fractures of the distal radius in children. In this small, unblinded series with no concurrent control group, all the 10 children enrolled completed the treatment, and no reasons to interrupt the study were encountered at any time. No skin lesions were reported at any time. Moreover, a positive response with regards to effect and emotional status was reported both from parents and children.

“Buckle” fractures of the distal radius were chosen for this study because they are relatively frequent and carry virtually no risk of secondary displacement or of cosmetic or functional consequences [26,27]. Limiting the application to this type of fracture may be regarded as excessively prudent and restrictive, but given the purpose of obtaining basic evaluation on patient comfort and satisfaction as well as on the practicability of the supply chain and the mechanical performance of the cast, it was felt that this limitation was excusable.

The general comfort received very low scores on the VAS scale, which confirmed the uneventful course of the disease.

No skin lesions according to NPUAP/EPUAP were reported at any time. Conclusions on the PANAS-C and the SAM scores are difficult to define. The results obtained with both scales did not show negative responses compared to normal normative scores and allowed hypothesising that the experience with the 3D device for wrist fractures of the participants was positive in terms of affect and emotional status.

The questionnaire that was submitted to children and parents highlighted consistent results throughout the study (Table 3). The average response to each question was quite unvarying and always in the positive range. Although not statistically significant, an overall better opinion of the cast was reported by the parents (Figure 6). This may be due to the fact that they were not personally injured and therefore limited, or to the ability to compare it to traditional casts because of either direct or indirect experience. It is understood that this questionnaire was not validated and was drawn up for the sole purpose of this study, so its scientific validity is questionable.

The clinical efficacy was likewise confirmed: the fitting of the cast was always considered satisfactory and deemed to offer adequate immobilisation. In the two cases where the cast displayed a wider tolerance to the metatarsal heads, it is thought that the problem arose from an imperfect fitting of the stockinette to the skin at the time of 3D scanning.

The clinical evaluation performed at the end of the treatment was uneventful, as expected for this type of fracture.

Clearly, the generally positive results of this study must be analysed, taking into account its distinct limitations. From the clinical standpoint, it is obvious that the condition that has been treated is very simple. We are also aware that the absence of a control group does not allow for a sound comparison of 3D printed casts to standard treatment. However, this study was meant to represent a starting point and was mainly conducted to assess the applicability of 3D patient-specific casts obtained with a specifically designed integrated system and to test its clinical safety. To properly address clinical outcomes, a prospective randomised trial to compare 3D printed patient-specific casts with standard treatment for wrist fractures in children is about to start at our institution. In a study on distal radius fractures in a mixed population of children and adults, data for the 3D-printed cast were obtained with CT scanning or MRI of both injured and uninjured limbs ten days after initial manipulation and standard cast application [16]. We believe that, while MRI is not always available, CT scanning for this purpose is not acceptable since it does not appear to satisfy the ALARA concept of exposure to radiation [28]. For this reason, we believe that the results in terms of ease of non-invasive acquisition and modelling, positive clinical results and soundness of the product, support further clinical investigation with this approach. The possibility of delayed 3D scanning (after first cast removal) at 10–15 days and subsequent 3D-printed cast application to complete treatment in more demanding fractures merits, however, further consideration.

Some of the tools implemented to quantify the wearability of the cast and the general comfort, specifically the PANAS-C, SAM and the patient/parent questionnaire, were applied for the first time or were specifically designed since other assessment instruments were not known to the authors or considered excessive. It is recognised that further confirmation is needed.

In this study, the definitive cast was printed in a facility outside the hospital, and products were made available, as reported, in the required timeframe. Nevertheless, it is felt that the possibility to have a 3D printer on-site would significantly streamline the procedure and, perhaps, reduce the already competitive price of the product.

## 5. Conclusions

The specifically designed integrated system (T3DDY-Oplà) proved to be simple to use and accurate in producing patient-specific 3D-printed casts that were safe and well-tolerated in children. In the ten consecutive patients enrolled in this study, pain was rarely reported and never exceeded VAS 3. No skin lesions according to the NPUAP/EPUAP classification were detected at any time. All answers in the evaluation questionnaire ranged in positive scores. In particular, high scores in the overall opinion of the cast were reported both by parents and children (4.7 and 4.2, respectively, on a Likert scale of 1 to 5). These latter scores well describe the positive acceptance of the device. Efficacy of contention fulfilled the expected tolerance measure of 3 mm in seven cases. No permanent deformations were noted in the used orthoses. The average measured error between the printed models and the reference models was 1 mm, with a maximum error of 2 mm. Although the casts were printed in an outside facility, all were available for application within 24 h of data acquisition. The results of this study constitute a valid basis on which to continue the experience. In fact, it is precisely the demonstrated feasibility and safety of patient-specific 3D printed casts that authorise the execution of a forthcoming randomised controlled trial that will compare these devices to standard treatment in more demanding fractures. Since the technology appears versatile and its costs may decrease in the near future, the identification of further potential applications in Orthopedics as in other specialties could nudge hospital institutions to consider 3D printers in their future budgets.

## Figures and Tables

**Table 1:** Pain by Visual Analog Scale (VAS) during treatment.

		VAS Results by Anatomic Landmark as in Figure 4									
Follow-up	Patient ID	A	B	C	D	E	F	H	I	L	M
**Day 3**	**4**	0	0	**1**	**1**	0	0	0	0	0	0
	**5**	0	0	**3**	0	0	0	0	0	0	0
**Day 21**	**2**	0	0		0	0	0	0	0	0	1
	**8**	0	0	**2**	0	0	0	**2**	0	0	0
	**10**	0	0	0	3	0	0	0	**1**	0	0

**Table 2:** Self-Assessment Manikin (SAM) results.

Domain	Day 3	Day 7	Day 21	Overall Mean (SD)
	Mean (SD)	Mean (SD)	Mean (SD)	
Pleasure	3.8 (±0.78)	3.8 (±1.22)	3.9 (±1.44)	3.9 (±1.06)
Arousal	1.3 (±0.70)	1.6 (±0.84)	2 (±0.66)	1.6 (±0.83)
Dominance	3.1 (±1.45)	3.7 (±0.94)	3.8 (±1.03)	3.6 (±1.09)

**Table 3:** General comfort questionnaire: scores are reported as means at each follow-up visit and as overall mean with SD.

	Day 3		Day 7		Day 21		Overall Mean (SD)	
Items	Child	Parents	Child	Parents	Child	Parents	Child	Parents
Have you been playing with your friends?	3.8	3.8	4.2	4	4.2	4	4.1 (0.6)	3.9 (0.5)
Did you shower as usual?	2.6	2.3	3.2	3.2	3.8	3.8	3.1 (0.7)	3.2 (0.6)
Did the cast limit your daily activities?	2.2	2.2	3.2	2.5	2.8	2.8	2.7 (0.9)	2.3 (0.9)
Did your arm itch?	1.7	1.3	2.6	1.6	2.1	2.1	2.2 (1.0)	1.5 (0.6)
Your opinion on the cast	3.9	4.8	4.3	4.7	4.5	4.5	4.2 (0.3)	4.7 (0.1)

**Table 4:** Maximum and minimum value of the deviation between the scanned models and the reference models and average of absolute values.

	Min Error (mm)	Max Error (mm)	Mean Deviation
Model 1	−0.58	0.64	0.915
Model 2	0.62	1.25	0.82
Model 3	−1.03	1.02	1.525
Model 4	0.87	2.02	1.355
Model 5	−1.22	1.84	0.865
Model 6	0.35	0.51	0.845
Model 7	0.74	1.34	1.33
Model 8	−1.77	1.92	1.36
Model 9	0.42	0.95	0.735
Model 10	0.98	1.05	0.98

## Data Availability

The data presented in this study are available on request from the corresponding author.

## Abbreviations

RE	Reverse Engineering
MRI	Magnetic Resonance Imaging
CT	Computed Tomography
DICOM	Digital Imaging and COmmunications in Medicine
VAS	Visual Analogue Scale
NPUAP	National Pressure Ulcer Advisory Panel
EPUAP	European Pressure Ulcer Advisory Panel
PANAS-C	Positive affect–Negative affect Scale for Children (PA: positive affect; NA: negative affect)
SAM	Self-Assessment Manikin
FDM	Fused Deposition Modeling
ABS	Acrylonitrile butadiene styrene
FDA	Food and Drug Administration
